# Electronic Smoking Devices Among University Students: Usage Patterns and Chemical Composition of Inhaled Substances

**DOI:** 10.1002/ansa.70059

**Published:** 2026-02-10

**Authors:** Eduard F. Valenzuela, Raffael Silva Santos Almeida, Ivana Ferreira Simões, Aline Gonçalves Miranda, Magno Oliveira Ramos, Roberto Rodrigues Bandeira Tosta Maciel, Fernanda Warken Rosa, Aníbal de Freitas Santos Júnior

**Affiliations:** ^1^ Departamento De Ciências da Vida (DCV) Universidade Do Estado da Bahia (UNEB) Salvador Bahia Brazil

**Keywords:** electronic cigarettes, nicotine dependence, public health, university students, volatile organic compounds

## Abstract

This study investigated the prevalence of electronic smoking device (ESD) use and its associated behavioural and chemical risks among university students in Bahia, Brazil. A cross‐sectional survey was conducted with 355 students from public and private institutions through an online questionnaire between April and May 2023. Among participants, 25.6% reported having previously used ESDs, and 11% used them daily, with dual use alongside conventional cigarettes being the most common pattern (61.5%). Most users were male (57.1%), single (95.2%) and reported low levels of nicotine dependence. Curiosity, peer influence and flavour variety were the main motivations for use. In parallel, chemical profiling was performed on a commonly consumed e‐liquid using DI‐SPME–GC–MS and in situ–SPME–GC–MS techniques. A total of 223 compounds were identified in the e‐liquid and 185 in the aerosol, including harmful substances, such as polycyclic aromatic hydrocarbons (PAHs), phthalic acids and fungicide dinocap. The samples were rich in terpenes, esters and solvents like glycerine and propylene glycol. The presence of toxic volatile and semi‐volatile organic compounds highlights potential health risks, especially under repeated exposure. These findings reinforce the urgency of implementing regulatory policies and preventive strategies focused on ESD use in academic environments.

## 1 Introduction

Tobacco use is a major public health problem responsible for over seven million deaths globally each year, including an estimated 1.6 million non‐smokers exposed to second‐hand smoke [1]. Despite public health efforts, more than one billion people continue to use tobacco products, with a prevalence of approximately 9.3% among Brazilian adults and a higher rate among men (11.7%) compared to women (7.2%) [2]. In recent years, electronic smoking devices (ESDs), also known as electronic cigarettes, vapes, pods or e‐hookahs, have gained widespread popularity, especially among young people. These devices deliver nicotine by heating a liquid solution or dry material, producing an aerosol that is inhaled by the user [3].

Despite a ban on sales in Brazil, their availability through informal markets and online stores has continued to increase [4]. In practice, the ban is poorly enforced, with e‐cigarettes readily sold near food establishments, in specialized vape shops and across digital platforms, often without any age verification mechanisms. Young people are particularly susceptible to marketing strategies and the sensory appeal of flavoured e‐liquids. According to a recent statistical study conducted in the United States, the most frequently reported reason for e‐cigarette use was coping with mental health issues (39.6%), followed by sensation seeking (20.4%), perceived reduced harm (14.7%), social status and acceptability (10.9%), ease of use and access (10.1%) and peer or family influence (4.3%) [5]. This perceived reduced harm is particularly ironic, as many users believe they can control or even overcome their addiction to conventional cigarettes (assumed to be more toxic) by switching to e‐cigarettes, thus supposedly reducing their nicotine exposure. However, studies have shown that commercial e‐liquids often contain high concentrations of nicotine, including products labelled ‘nicotine‐free’, raising concerns about misleading marketing and the potential for sustained or increased dependence [6, 7].

E‐liquids are complex chemical mixtures primarily composed of propylene glycol and glycerol, which serve as solvents and vapour carriers. Nicotine, either in freebase or salt form, is typically present in varying concentrations, depending on the product. In addition to these primary components, e‐liquids may contain flavouring agents, such as flavonoids, terpenes, aldehydes and alcohols, many of which are generally recognized as safe (GRAS) for ingestion but not necessarily for inhalation [8, 9, 10]. However, several studies have identified the presence of potentially harmful substances in commercially available e‐liquids. These include heavy metals (e.g., lead, chromium and nickel) [11, 12, 13], tobacco‐specific nitrosamines (TSNAs) [14, 15], polycyclic aromatic hydrocarbons (PAHs) [16] and volatile organic compounds (VOCs), such as benzene, toluene, ethylbenzene and xylene (BTEX) [17]. Vitamin E acetate, used as a thickening agent particularly in THC‐containing vape products, has also been implicated in respiratory toxicity [18]. The thermal degradation of some components during vaporization can further increase the formation of reactive aldehydes and other toxic intermediates, posing additional health risks to users [19].

The inhalation of toxic substances present in e‐liquids has been associated with a range of adverse health outcomes. Respiratory diseases, such as e‐cigarette or vaping product use‐associated lung injury (EVALI), bronchiolitis obliterans (popcorn lung) and acute lipoid pneumonia, have been reported, particularly in cases involving vitamin E acetate or lipid‐based carriers [20, 21]. Chronic exposure to aldehydes, BTEX compounds and metals may also contribute to oxidative stress, inflammation and damage to pulmonary tissue, potentially leading to chronic obstructive pulmonary disease (COPD), asthma exacerbation, cardiovascular complications and cancer risks [22, 23, 24]. Furthermore, nitrosamines and PAHs are known carcinogens, raising concerns about long‐term cancer risks associated with sustained vaping use [25, 26]. Cardiovascular toxicity and neurological symptoms, such as headaches, dizziness and cognitive impairment, have also been described, possibly linked to nicotine overdose or exposure to neurotoxic solvents [27, 28].

SPME is a well‐established analytical technique that has been widely used for over three decades in the detection of volatile and semi‐volatile compounds in different matrices [29, 30]. In the context of ESDs or mainstream cigarettes, SPME coupled with GC–MS has proven to be a powerful tool for identifying toxic substances present in aerosols and e‐liquids [10, 31]. Studies have shown that this method effectively captures harmful compounds, such as aldehydes, alcohols, aromatic hydrocarbons and other VOCs generated during vaporization [32, 33]. Its solvent‐free nature, high sensitivity and operational simplicity make SPME particularly valuable for assessing the chemical profile of inhaled emissions and for comparing declared ingredients with real‐world exposures [34].

The present study is structured in two complementary parts. The first aims to estimate the prevalence of ESD use and to describe the sociodemographic and behavioural profile of university students in Salvador, Bahia, Brazil. The second part seeks to enhance the analysis by chemically characterizing the VOCs and SVOCs (semi‐volatile organic compounds) inhaled by these users, using SPME–GC–MS. To this end, a commercially available e‐liquid commonly used by local ESD consumers was analysed to identify volatile and semi‐volatile toxic compounds released during vaporization. Together, these approaches provide both epidemiological and chemical perspectives on ESD consumption and its potential health risks. This is the first study conducted in the state of Bahia to integrate behavioural data with chemical analysis of both the liquid and aerosol.

## 2 Methods

### 2.1 Study Design and Population

A cross‐sectional, descriptive study was conducted with a convenience sample of 355 students from public and private universities in Salvador, Bahia, Brazil. Eligibility criteria included current enrolment in an undergraduate or graduate program and providing informed consent to participate in the study. Data were collected between April and May 2023, using a structured online questionnaire disseminated through WhatsApp, Instagram, academic leagues and student associations (Supporting Information section). A pilot test was performed with students from the State University of Bahia (UNEB) to validate the questionnaire's clarity and relevance. The questionnaire included sociodemographic information (gender, age, race, marital status, income, academic program, institution type and living situation), behavioural data (alcohol consumption, tobacco use) and ESD‐specific variables (frequency, duration, nicotine concentration, flavours, reasons for use and points of access). Nicotine dependence was measured using the Penn State Nicotine Dependence Index (PSNDI), a 10‐item instrument with scores ranging from 0 to 20. Dependence was classified as 0–3 (none), 4–8 (mild), 9–12 (moderate) and ≥13 (high). Data were analysed using SPSS Statistics version 17.0. Descriptive statistics included means, standard deviations and proportions.

### 2.2 Chemical Profile of VOCs and SVOCs

#### 2.2.1 Analytical Conditions for SPME–GC–MS Profiling

VOCs and SVOCs were analysed using a gas chromatograph (Agilent 7890C) coupled to a quadrupole mass spectrometer (Agilent 5977C), both manufactured in Palo Alto, CA, USA, and equipped with an automated sample handling system (PAL RSI 85). Chromatographic separation was achieved using two HP‐5MS columns (Agilent J&W GC Columns, 15 m × 250 µm × 0.25 µm) connected in series. The oven temperature program began at 40°C, increased to 100°C at a rate of 5°C/min (held for 2 min), followed by a ramp to 270°C at 15°C/min (held for 10 min), for a total run time of 35.33 min. Helium was used as the carrier gas at a constant flow rate of 1.0 mL/min. The injector was operated in splitless mode at 250°C. Mass spectrometry was performed in scan mode over a mass range of 35–550 *m*/*z*, with a solvent delay of 1 min. The ion source and quadrupole temperatures were set at 230°C and 150°C, respectively.

Compound identification was based on spectral matching against the NIST 2020 Mass Spectral Library (version 2.4), using a minimum match factor of 50. For the resolution of co‐eluted analytes and spectral deconvolution, the MassHunter Unknowns Analysis software (Agilent, version 10.2) was employed, allowing improved accuracy in peak identification. This identification was supported by a relative standard deviation (RSD) below 5.5%, calculated from the peak areas of three analytical replicates, as an indicator of repeatability. This calculation considered only those peaks with areas exceeding 50,000 a.u., which corresponded to well‐defined signals exhibiting signal‐to‐noise ratios (S/N) greater than 100. This ensures that the reported peak areas represent true analytical signals and are not confused with background noise.

Sample preconcentration was carried out using SPME with a DVB/PDMS/Carbon WR Smart SPME Fiber (80 µm total thickness: 50 µm/30 µm) from Agilent Technologies.

#### 2.2.2 Sample Preparation and Extraction Procedures

**FIGURE 1:**
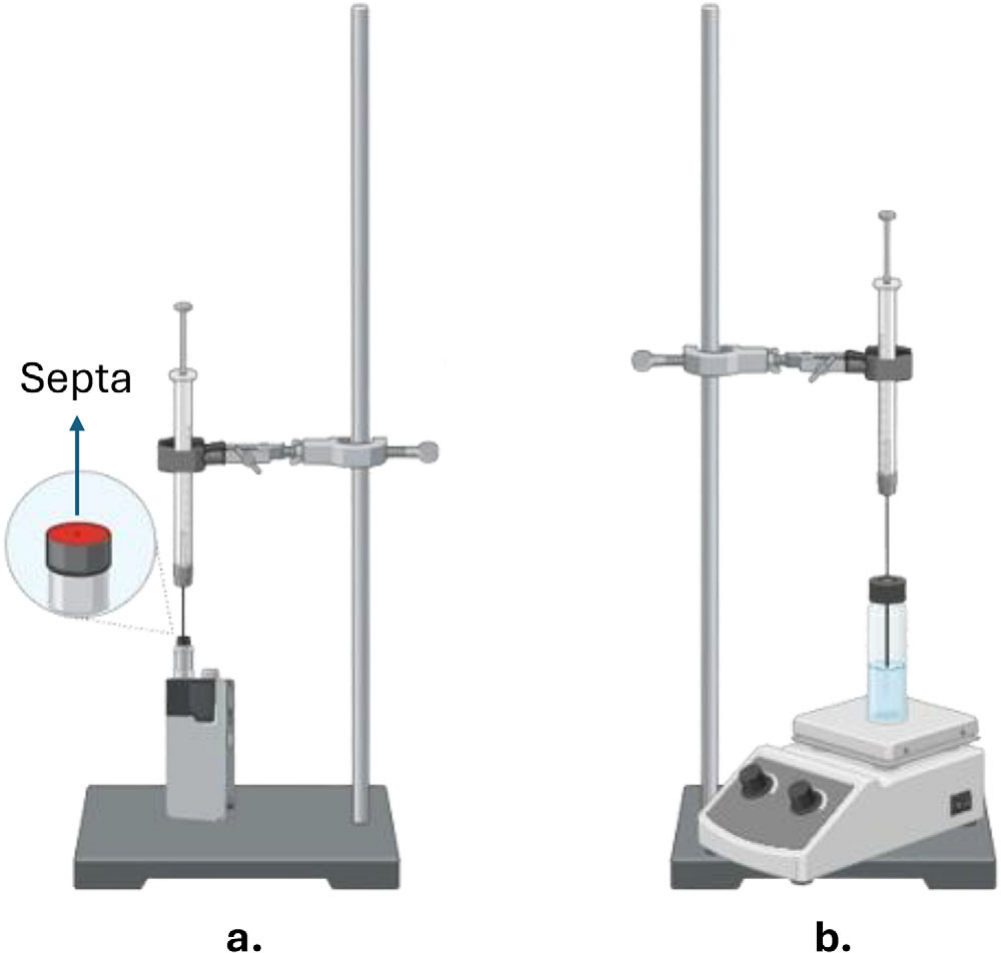
Schematic representation of the sample preparation and extraction procedures used in this study: (a) in situ–SPME configuration for aerosol sampling; (b) DI‐SPME for the analysis of e‐liquid sample.

For the analysis of the e‐liquid, a sample volume of 1.0 mL was transferred into a 20.0 mL headspace vial containing 1.0 g of sodium chloride, 9.0 mL of Type 1 water and a magnetic stirring bar. The mixture was gently stirred at 250 rpm using a magnetic stirrer to dissolve the salt, enhancing the salting‐out effect. The extraction process was carried out at room temperature. The extraction of VOCs and SVOCs was then performed using DI‐SPME for 5 min prior to injection into the GC–MS system (Figure 1a). Key SPME parameters, such as extraction mode (DI vs. HS), temperature, salting‐out effect, agitation and extraction time, were adjusted on the basis of previous studies applying this technique to complex matrices [30, 35, 36].

For the aerosol analysis, an in situ–SPME approach was employed. Leveraging the structural design of the e‐cigarette, the device was sealed by placing a gas chromatography septum on its rear opening, effectively creating a closed system and allowing the SPME fibre to be introduced internally for compound capture and preconcentration (Figure 1b). Once the fibre was inserted, three vaporization puffs (resistor activation) were initiated, simulating real use. The fibre remained exposed for 5 min before being retracted and thermally desorbed into the GC–MS injector.

#### 2.2.3 Sample Description and Acquisition

The sample analysed in this study was a commercial e‐liquid purchased in Brazil via the Mercado Livre platform. It is a product from the brand *APEX Resistance Fuel*, labelled ‘Strength Aromatic Pipe Tobacco with tropical fruit finish’, with a net content of 30 mL. According to the label, the formulation contains zero nicotine (0 mg/mL), a precaution often taken by users aiming to reduce nicotine exposure. This sample was selected as a representative of a commonly consumed product in the local urban market.

### 2.3 Ethical Considerations

The study was approved by the Research Ethics Committee of the State University of Bahia (CAAE: 56061622.4.0000.0057). All participants provided electronic informed consent prior to participation.

## 3 Results and Discussion

### 3.1 Behavioural and Sociodemographic Patterns

This study included 355 university students from Salvador, Bahia, Brazil, of whom 91 (25.6%) reported having used ESDs at some point. Among tobacco product users, dual use, defined as the combined use of electronic and conventional cigarettes, was the most prevalent pattern (61.5%), followed by exclusive ESD use (38.5%). The sociodemographic and behavioural characteristics of participants are presented in Table 1, along with data on experimentation and current use of tobacco products.

**TABLE 1:** Frequency of experimentation and use of electronic smoking devices (ESDs) and dual use, according to sociodemographic characteristics of 91 university students in Salvador, Bahia, Brazil—2023.

Sociodemographic characteristics	Total *n* (%)	ESD use *n* (%)	Dual use *n* (%)	*p* value
**Gender**				
Female	55 (60.4)	23 (25.3)	32 (35.2)	0.278
Male	36 (39.6)	12 (13.2)	24 (26.4)	
**Race/Ethnicity**				
White	41 (45.1)	17 (18.7)	24 (26.4)	0.375
Black or Brown	50 (54.9)	18 (19.8)	32 (35.2)	
**Marital status**				
Single	85 (93.4)	32 (35.2)	53 (58.2)	0.423
Married	6 (6.6)	3 (3.3)	3 (3.3)	
**Household income (in minimum wages** ^a^)				
≤1–3 (≤BRL 3906.00)	41 (45.1)	14 (15.4)	27 (29.7)	0.021
>3–9 (BRL 3906.01–11,718.00)	36 (39.6)	11 (12.1)	25 (27.5)	
≥10 (≥BRL 13,020.00)	14 (15.4)	10 (11.0)	4 (4.4)	
**Living arrangement**				
Alone	8 (8.8)	3 (3.3)	5 (5.5)	0.921
With parents or guardians	71 (78.0)	28 (39.4)	43 (47.3)	
With friends or partner	12 (13.2)	4 (4.4)	8 (8.8)	
**Current educational level**				
Undergraduate	87 (95.6)	34 (37.4)	53 (58.2)	0.531
Graduate	4 (4.4)	1 (1.1)	3 (3.3)	
**Type of higher education institution**				
Public	42 (46.2)	16 (38.1)	26 (61.9)	0.560
Private	49 (53.8)	19 (38.8)	30 (61.2)	
**Field of study**				
Health sciences	45 (49.5)	22 (24.2)	23 (25.3)	0.240
Exact sciences and engineering	18 (19.8)	5 (5.5)	13 (14.3)	
Humanities and arts	16 (17.6)	5 (5.5)	11 (12.1)	
Agrarian/Biological sciences	12 (13.2)	3 (3.3)	9 (9.9)	
**Frequency of ESD use**				
Daily use	10 (11.0)	5 (5.5)	5 (5.5)	0.664
Less than daily (e.g., weekends)	17 (18.7)	7 (7.7)	10 (11.0)	
Former user	64 (70.3)	35 (25.2)	41 (45.1)	
**Alcohol consumption**				
Yes	77 (84.6)	29 (31.9)	48 (52.7)	0.713
No	14 (15.4)	6 (6.6)	8 (8.8)	
**Age (years)**	Mean ± SD	—	—	
	22.0 ± 2.8			0.250
**Total**	91 (100)	35 (38.5)	56 (61.5)	

Abbreviations: ESD, electronic smoking device; SD, standard deviation.

^a^Pearson's Chi‐square test. Minimum wage in effect on 01/01/2023: BRL 1302.00.

The sample had a mean age of 22 ± 2.8 years, with a predominance of female participants (60.4%), self‐identified Black or mixed‐race individuals (53.8%) and single students (93.4%). Most students lived with family members (78%) and reported household income between three and nine minimum wages. A total of 95.6% were enrolled in undergraduate programs, primarily at private institutions (53.8%). Health science students were the most represented (49.5%), followed by those in engineering or exact sciences (19.8%) and humanities (17.6%).

**FIGURE 2:**
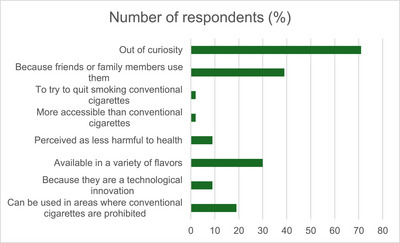
Reported reasons for using electronic smoking devices among university students (Salvador, Brazil, 2023).

**FIGURE 3:**
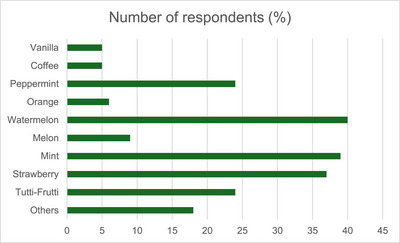
Frequency distribution of preferred e‐liquid flavours among university students who use electronic smoking devices (Salvador, Brazil, 2023).

Among all ESD users, most had used the devices in the past (70.3%), with 18 participants (18.7%) using them less than daily, and 10 participants (11%) reported daily use. Notably, female students showed higher proportions of both exclusive ESD use (25.3%) and dual use (35.2%). The main reasons cited for initiating ESD use included curiosity (78%) and peer or family influence (42.9%) (Figure 2). The most frequently used e‐liquid flavours were watermelon (44%), mint (42.9%) and strawberry (40.7%) (Figure 3).

Focusing on current users (*n* = 21), their average age was 21.8 ± 2.7 years; overall, 57.1% were male, 47.6% identified as white and 95.2% were single (Table 2). Approximately half (47.8%) reported a family income between one and three minimum wages, 28.6% between three and nine and 28.6% above 10 minimum wages. One‐third (33.3%) lived with smokers, 90.5% attended private universities and 90.5% also reported alcohol consumption. Regarding nicotine use, 52.4% used ESDs daily and 47.6% had less than 1 year of use. Most participants consumed nicotine concentrations between 3.1 and 5.0 mg/mL (52.4%), whereas 23.8% reported using products with concentrations above 20 mg/mL. Mild nicotine dependence was most common (33.3%), although 38% showed moderate‐to‐high dependence.

**TABLE 2:** Frequency of current electronic smoking device (ESD) use with nicotine according to sociodemographic characteristics of 21 university students in Salvador, Bahia, Brazil—2023.

Sociodemographic characteristics	*n* = 21	%
**Gender**		
Female	9	42.9
Male	12	57.1
**Race/Ethnicity**		
White	10	47.6
Brown	7	33.3
Black	4	19.0
**Marital status**		
Single	20	95.2
Married	1	4.8
**Household income (in minimum wages** ^a^)		
≤1–3 (≤BRL 3906.00)	9	47.8
>3–9 (BRL 3906.01–11,718.00)	6	28.6
≥10 (≥BRL 13,020.00)	6	28.6
**Living arrangement**		
With parents or guardians	15	71.4
With friends or partner	4	19.0
Alone	2	9.5
**Lives with someone who smokes**		
Yes	7	33.3
No	14	66.7
**Current educational level**		
Undergraduate	19	90.5
Graduate	2	9.5
**Type of higher education institution**		
Private	15	71.4
Public	6	28.6
**Alcohol consumption**		
Yes	19	90.5
No	2	9.5
**Frequency of ESD use**		
Daily (every day)	11	52.4
Less than daily (e.g., weekends)	10	47.6
**Duration of ESD use (in years)**		
<1 year	10	47.6
1–5 years	11	52.4
**Nicotine concentration consumed**		
Unknown	1	4.8
1.0–3.0 mg/mL	3	14.3
3.1–5.0 mg/mL	11	52.4
5.1–10.0 mg/mL	1	4.8
More than 20.0 mg/mL	5	23.8
**Nicotine dependence (based on e‐cigarette use)** ^b^		
None	6	28.6
Mild	7	33.3
Moderate	4	19.0
High	4	19.0
**Main reasons for using ESDs**		
Can be used in smoke‐free areas	12	57.1
Out of curiosity	11	52.4
Available in various flavours	11	47.6
Friends or family members use them	9	42.9
Perceived as less harmful to health	6	28.6
Considered a technological innovation	5	23.8
To help quit traditional cigarettes	2	9.5
More affordable than conventional cigarettes	1	4.8
**Flavour preferences**		
Strawberry	13	61.9
Mint	12	57.1
Menthol	12	57.1
Watermelon	9	42.9
Tutti‐frutti	6	28.6
Other flavours^c^	13	61.9
**Places of purchase/access**		
Physical stores (national)	13	61.9
Online stores (national)	9	42.9
Street vendors	5	23.8
Gift from friends or family	2	9.5
International physical/online store	2	9.5
**Mean age (±SD)**	21.8 ± 2.7	

Abbreviation: ESD, electronic smoking device.

^a^Minimum wage in effect on 01/01/2023: BRL 1302.00.

^b^Based on the Penn State Nicotine Dependency Index.

^c^Other flavours reported: vanilla, orange, mango, blueberry ice, peach, grape, green apple, tangerine.

Flavour variety played an important role in user preferences: The most reported were strawberry (61.9%), mint (57.1%), menthol (57.1%) and watermelon (42.9%). Multiple other flavours were also mentioned by 61.9% of participants. Regarding access, physical retail stores were the primary source (61.9%), followed by national online shops (42.9%) and street vendors (23.8%).

These findings mirror trends in other regions of Brazil and internationally. Studies in Saudi Arabia, France and the United States have similarly documented rising ESD use among young adults [37, 38, 39]. Notably, previous studies in Brazil reported lower usage rates. For example, Oliveira et al. [40] found experimentation at 2.7% and current use at only 0.61%, indicating a possible acceleration in ESD uptake due to increased availability and misconceptions about safety.

The predominance of female ESD users aligns with findings by Castro et al., suggesting a convergence of social, cultural, and risk perception factors [41]. Family income appeared to influence ESD access: Higher‐income students were more likely to afford advanced or flavoured devices, supporting international data indicating affordability as a barrier to ESD use [42, 43, 44]. The curiosity‐driven initiation and the influence of social networks were consistent with studies by Romijnders et al. and Kurdi et al., emphasizing the role of social norms and peer modelling [45, 46]. The ability to use ESDs in indoor or restricted environments and the appeal of diverse flavours are also major motivators, especially in this age group. Recent evidence has associated flavoured ESDs, particularly mint, with impaired pulmonary function [47], and flavoured products are often perceived as less harmful [48].

The present study also highlighted nicotine exposure patterns. Although many participants used low to moderate concentrations, a significant proportion reported use of highly concentrated products (>20 mg/mL), corroborating studies indicating that ESDs can deliver nicotine at levels comparable to or exceeding conventional cigarettes [49, 50]. These exposure levels pose substantial risks for addiction, particularly among nicotine‐naïve users. Goldenson et al. also found a considerable proportion of US adolescents using high‐nicotine e‐liquids, paralleling the trends observed here [51]. Finally, several limitations should be acknowledged. The use of a convenience sample restricts generalizability. Social desirability bias and non‐response among ESD users may have led to underestimation. Nevertheless, the data offer important insights into a population at elevated risk for nicotine addiction and exposure to harmful substances.

### 3.2 Chemical Characterization of E‐Liquid and Aerosol Composition

The combined chemical analysis of both the e‐liquid and aerosol samples using DI‐SPME–GC–MS and in situ–SPME–GC–MS, respectively, led to the identification of 375 VOCs and SVOCs. Specifically, 223 compounds were detected in the e‐liquid and 185 in the aerosol, with only 33 compounds shared between both matrices. Compound identification was based on match factors greater than 50% against the NIST 2020 version 2.4 database, with most matches exceeding 75%, ensuring a high level of confidence in the results. The identified compounds span diverse chemical families, including alcohols, esters, carboxylic acids, ketones, terpenes, phenolics, aliphatic and aromatic hydrocarbons as well as nitrogen‐ and oxygen‐containing compounds. This chemical diversity reflects the complex formulation of e‐liquids, which includes both intended ingredients (e.g., solvents and flavour additives) and possible contaminants introduced during manufacturing, storage or thermal degradation. These findings are consistent with previous studies reporting high chemical complexity in vaping products [52, 53, 54]. Notably, although this study did not employ the enhanced separation capabilities of comprehensive two‐dimensional gas chromatography (GC × GC), the use of convolution processing techniques during data analysis enabled the identification of many compounds, underscoring the robustness of the analytical approach. The full list of identified compounds is provided in Table 3.

**TABLE 3:** Comparative chemical profile of volatile organic compounds (VOCs) and semi‐volatile organic compounds (SVOCs) detected in e‐liquid (DI‐SPME) and aerosol (in situ–SPME) from a commercially available nicotine‐free electronic smoking device (ESD) formulation.

#	RT	Compound name	Match factor	Formula	CAS#	E‐liquid	Aerosol
1	1.48	Dimethyl ether	94	C_2_H_6_O	115‐10‐6	✓	✓
2	1.56	Ethyl acetoxy(acetyl)carbamate	63	C_7_H_11_NO_5_	1000385‐74‐8		✓
3	1.89	Propanoic acid, butyl ester	81	C_7_H_14_O_2_	590‐01‐2	✓	
4	1.91	Hydroxyurea	68	CH_4_N_2_O_2_	127‐07‐1		✓
5	1.91	Acetic acid, dimethoxy‐, methyl ester	90	C_5_H_10_O_4_	89‐91‐8		✓
6	1.96	*n*‐Hexane	96	C_6_H_14_	110‐54‐3	✓	
7	2.05	Ethyl acetate	96	C_4_H_8_O_2_	141‐78‐6	✓	
8	2.13	Propane, 1‐isocyanato‐	74	C_4_H_7_NO	110‐78‐1	✓	
9	2.31	sec‐Butylamine	84	C_4_H_11_N	13952‐84‐6	✓	
10	2.40	2‐Propenal	87	C_3_H_4_O	107‐02‐8		✓
11	2.40	2‐Propenoic acid, 2‐propenyl ester	61	C_6_H_8_O_2_	999‐55‐3		✓
12	2.41	1‐Butanol, 2‐amino‐	56	C_4_H_11_NO	96‐20‐8		✓
13	2.48	2‐Propanol, 1‐methoxy‐	80	C_4_H_10_O_2_	107‐98‐2	✓	
14	2.51	Acetohydroxamic acid	85	C_2_H_5_NO_2_	546‐88‐3		✓
15	2.66	1,3‐Dioxane, 2‐methyl‐	92	C_5_H_10_O_2_	626‐68‐6	✓	✓
16	2.85	*n*‐Propyl acetate	89	C_5_H_10_O_2_	109‐60‐4	✓	
17	3.08	1‐Pentanol	96	C_5_H_12_O	71‐41‐0	✓	
18	3.12	Furfural	52	C_5_H_4_O_2_	98‐01‐1		✓
19	3.37	Glycidol	78	C_3_H_6_O_2_	556‐52‐5	✓	
20	3.38	Propylene glycol	97	C_3_H_8_O_2_	57‐55‐6	✓	✓
21	3.38	Trimethylphosphine	66	C_3_H_9_P	594‐09‐2	✓	
22	3.54	Toluene	86	C_7_H_8_	108‐88‐3	✓	
23	3.60	Acetic acid, methyl ester	50	C_3_H_6_O_2_	79‐20‐9		✓
24	3.80	1,3‐Dioxolane, 2‐ethyl‐4‐methyl‐	90	C_6_H_12_O_2_	4359‐46‐0	✓	
25	4.02	1‐Butanamine, *N*,*N*‐dimethyl‐	62	C_6_H_15_N	927‐62‐8		✓
26	4.02	Pentanoic acid, 2‐methyl‐, methyl ester	61	C_7_H_14_O_2_	2177‐77‐7		✓
27	4.08	Butanoic acid, ethyl ester	97	C_6_H_12_O_2_	105‐54‐4	✓	
28	4.19	Methane, (methylsulfinyl)(methylthio)‐	57	C_3_H_8_OS_2_	33577‐16‐1		✓
29	4.79	1,3‐Propanediol	93	C_3_H_8_O_2_	504‐63‐2	✓	
30	4.83	Nitric acid, ethyl ester	67	C_2_H_5_NO_3_	625‐58‐1		✓
31	4.87	Methyl 2‐hydroxy‐2‐methoxyacetate	80	C_4_H_8_O_4_	19757‐97‐2		✓
32	4.90	1,3‐Dioxolane, 4‐methyl‐2‐propyl‐	92	C_7_H_14_O_2_	4352‐99‐2	✓	
33	4.93	3‐Hexanol, 2,3‐dimethyl‐	58	C_8_H_18_O	4166‐46‐5		✓
34	5.05	Butanoic acid, 2‐ethyl‐2,3,3‐trimethyl‐	68	C_9_H_18_O_2_	38541‐67‐2	✓	
35	5.09	3‐Hexen‐1‐ol, formate, (*Z*)‐	84	C_7_H_12_O_2_	33467‐73‐1	✓	
36	5.12	5‐Heptenal, 2,6‐dimethyl‐	80	C_9_H_16_O	106‐72‐9		✓
37	5.17	3‐Hexen‐1‐ol, (*Z*)‐	99	C_6_H_12_O	928‐96‐1	✓	
38	5.21	1H‐Imidazole‐4‐methanol	71	C_4_H_6_N_2_O	822‐55‐9		✓
39	5.62	Neopentylamine	59	C_5_H_13_N	5813‐64‐9		✓
40	5.64	1‐Butanol, 3‐methyl‐, acetate	99	C_7_H_14_O_2_	123‐92‐2	✓	✓
41	5.75	1,2‐Propanediol, 1‐acetate	93	C_5_H_10_O_3_	627‐69‐0	✓	
42	5.90	Benzene, 1,1′‐(1,2‐cyclobutanediyl)bis‐, *cis*‐	73	C_16_H_16_	7694‐30‐6		✓
43	5.95	2‐Heptanone	94	C_7_H_14_O	110‐43‐0	✓	
44	5.96	Butanedioic acid, phenyl‐	87	C_10_H_10_O_4_	635‐51‐8	✓	✓
45	6.08	1,2‐Propanediol, 2‐acetate	87	C_5_H_10_O_3_	—	✓	
46	6.17	Urea, methyl‐	74	C_2_H_6_N_2_O	598‐50‐5		✓
47	6.22	2‐Acetamido‐*N*‐methylacetamide	75	C_5_H_10_N_2_O_2_	7606‐79‐3		✓
48	6.34	Oxime‐, methoxy‐phenyl‐	67	C_8_H_9_NO_2_	1000222‐86‐6	✓	
49	6.44	Oxazolidin‐2‐one	77	C_3_H_5_NO_2_	497‐25‐6		✓
50	6.45	1,2,5‐Trimethylpyrrole	67	C_7_H_11_N	930‐87‐0	✓	
51	6.45	Pyrazine, 2,6‐dimethyl‐	80	C_6_H_8_N_2_	108‐50‐9	✓	
52	7.03	Desmethyldeprenyl	67	C_12_H_15_N	18913‐84‐3	✓	
53	7.16	Proline, 2‐methyl‐5‐oxo‐, methyl ester	78	C_7_H_11_NO_3_	56145‐24‐5		✓
54	7.25	1,3‐Dioxolane, 4‐methyl‐2‐(2‐methylpropyl)‐	95	C_8_H_16_O_2_	18433‐93‐7	✓	✓
55	7.41	1,3‐Dioxane	79	C_4_H_8_O_2_	505‐22‐6	✓	
56	7.59	5‐Fluoro‐2‐trifluoromethylbenzoic acid, 2‐formyl‐4,6‐dichlorophenyl ester	68	C_15_H_6_Cl_2_F_4_O_3_	1000331‐61‐0		✓
57	7.59	1,3‐Dioxane, 2‐heptyl‐	63	C_11_H_22_O_2_	5702‐44‐3	✓	
58	7.73	Benzaldehyde	98	C_7_H_6_O	100‐52‐7	✓	
59	7.83	2‐Furancarboxaldehyde, 5‐methyl‐	88	C_6_H_6_O_2_	620‐02‐0	✓	
60	7.85	1,2‐Propanediol, 3‐chloro‐	95	C_3_H_7_ClO_2_	96‐24‐2		✓
61	8.01	Disulphide, propyl 1‐(propylthio)ethyl	64	C_8_H_18_S_3_	69078‐86‐0	✓	
62	8.11	Bromonitromethane	66	CH_2_BrNO_2_	563‐70‐2		✓
63	8.11	5‐Acetyl‐2‐amino‐4‐methylpyrimidine	66	C_7_H_9_N_3_O	66373‐25‐9		✓
64	8.29	Diglycolic acid, pentyl phenethyl ester	63	C_17_H_24_O_5_	1010382‐15‐9	✓	
65	8.51	Benzenecarboximidoyl bromide, *N*‐methyl‐	56	C_8_H_8_BrN	41182‐85‐8		✓
66	8.59	Ethanone, 1‐cyclopropyl‐2‐(4‐pyridinyl)‐	62	C_10_H_11_NO	6580‐95‐6	✓	
67	8.77	Methyl methylphosphonofluoridate	51	C_2_H_6_FO_2_P	353‐88‐8	✓	
68	8.79	9,10‐Anthracenedione, 1,3‐dihydroxy‐4‐methoxy‐2‐methyl‐	55	C_16_H_12_O_5_	34155‐88‐9		✓
69	8.79	1,2‐Benzenediol, *O*‐(2‐furoyl)‐*O*′‐(pentafluoropropionyl)‐	60	C_14_H_7_F_5_O_5_	1010329‐74‐7	✓	
70	8.84	Propanoic acid, anhydride	60	C_6_H_10_O_3_	123‐62‐6	✓	
71	8.84	Hexanoic acid, ethyl ester	96	C_8_H_16_O_2_	123‐66‐0	✓	✓
72	8.87	Pyrazine, trimethyl‐	90	C_7_H_10_N_2_	14667‐55‐1	✓	✓
73	8.87	2‐*n*‐Butyl furan	62	C_8_H_12_O	4466‐24‐4	✓	
74	8.92	Cyclopentanecarboxylic acid, 2‐fluorophenyl ester	67	C_12_H_13_FO_2_	1000325‐76‐5	✓	✓
75	8.96	l‐Alanine, *N*‐(2‐furoyl)‐, ethyl ester	71	C_10_H_13_NO_4_	1000314‐28‐0	✓	
76	9.05	3‐Hexen‐1‐ol, acetate, (*Z*)‐	97	C_8_H_14_O_2_	3681‐71‐8	✓	✓
77	9.36	Thiazole, 4‐methyl‐2‐(1‐methylethyl)‐	89	C_7_H_11_NS	15679‐13‐7	✓	
78	9.40	Glycine, methyl ester	55	C_3_H_7_NO_2_	616‐34‐2		✓
79	9.41	Acetylpyrazine	93	C_6_H_6_N_2_O	22047‐25‐2	✓	
80	9.56	Cyclobutane, 1,3‐diisopropenyl‐, *trans*	78	C_10_H_16_	1000152‐89‐6		✓
81	9.64	d‐limonene	92	C_10_H_16_	5989‐27‐5	✓	✓
82	9.67	1‐Hexanol, 2‐ethyl‐	66	C_8_H_18_O	104‐76‐7	✓	
83	9.67	1‐Pentanol, 2‐ethyl‐4‐methyl‐	67	C_8_H_18_O	106‐67‐2	✓	
84	9.77	Ethanone, 1‐(2‐pyridinyl)‐	95	C_7_H_7_NO	1122‐62‐9	✓	✓
85	9.78	Methanesulphonyl chloride	60	CH_3_ClO_2_S	124‐63‐0	✓	✓
86	9.79	Ethene, chloro‐	86	C_2_H_3_Cl	75‐01‐4		✓
87	9.79	2‐Propynoic acid, methyl ester	54	C_4_H4O_2_	922‐67‐8	✓	
88	9.82	Carbamic acid, methyl‐, 3‐methylphenyl ester	79	C_9_H_11_NO_2_	1129‐41‐5	✓	
89	9.83	2,4(1*H*,3*H*)‐Pyrimidinedione, 1,3‐dimethyl‐	64	C_6_H_8_N_2_O_2_	874‐14‐6	✓	
90	9.83	1,2‐Propanediol, 1‐phenyl‐	79	C_9_H_12_O_2_	1855‐09‐0		✓
91	9.85	4‐Methylphenyl beta‐phenylpropionate	60	C_16_H_16_O_2_	22020‐95‐7		✓
92	9.92	2‐Propanol, 1,1′‐oxybis‐	87	C_6_H_14_O_3_	110‐98‐5		✓
93	10.07	Benzeneacetaldehyde	87	C_8_H_8_O	122‐78‐1	✓	
94	10.22	1,3,5‐Trioxane	84	C_3_H_6_O_3_	110‐88‐3		✓
95	10.39	2,2‐Dimethyl‐3‐heptanone	63	C_9_H_18_O	19078‐97‐8		✓
96	10.53	(*E*)‐2,6‐Dimethylocta‐3,7‐diene‐2,6‐diol	56	C_10_H_18_O_2_	51276‐34‐7	✓	
97	10.61	1,2‐Ethanediol, diformate	58	C_4_H_6_O_4_	629‐15‐2		✓
98	10.72	1‐Hexanone, 5‐methyl‐1‐phenyl‐	85	C_13_H_18_O	25552‐17‐4	✓	
99	10.79	(*Z*)‐Cinnamyl benzoate	59	C_16_H_14_O_2_	117204‐78‐1		✓
100	10.92	2‐Furanmethanol, 5‐ethenyltetrahydro‐.alpha, alpha, 5‐trimethyl‐, *cis*‐	70	C_10_H_18_O_2_	5989‐33‐3	✓	
101	10.94	1‐[(1‐Propoxypropan‐2‐yl)oxy]propan‐2‐yl acetate	72	C_11_H_22_O_4_	1000378‐33‐1	✓	
102	10.94	Dipropylene glycol, diacetate	79	C_10_H_18_O_5_	1000506‐25‐8	✓	
103	11.09	*o*‐Anisic acid, cyclobutyl ester	75	C_12_H_14_O_3_	1000299‐94‐6		✓
104	11.09	4′‐Butoxy‐2′‐methylacetophenone	73	C_13_H_18_O_2_	1000195‐98‐2		✓
105	11.20	Furan, 2,5‐dihydro‐2,5‐dimethoxy‐	68	C_6_H_10_O_3_	332‐77‐4	✓	
106	11.20	2‐Chloroaniline‐5‐sulfonic acid	65	C_6_H_6_ClNO_3_S	98‐36‐2	✓	
107	11.25	Pyrazine, 3‐ethyl‐2,5‐dimethyl‐	90	C_8_H_12_N_2_	13360‐65‐1	✓	
108	11.26	Benzene, (3‐iodo‐1‐methoxy‐1‐methylpropyl)‐	62	C_11_H_15_IO	1010327‐38‐7		✓
109	11.26	2‐Hexanol, 3,3,5‐trimethyl‐2‐(3‐methylphenyl)‐	59	C_16_H_26_O	274266‐33‐0		✓
110	11.31	Pyrazine, tetramethyl‐	78	C_8_H_12_N_2_	1124‐11‐4	✓	
111	11.33	4,4′‐Bitriazolyl	65	C_4_H_4_N_6_	16227‐15‐9		✓
112	11.37	beta‐Myrcene	65	C_10_H_16_	123‐35‐3	✓	
113	11.40	Phenol, 2‐methoxy‐	83	C_7_H_8_O_2_	90‐05‐1	✓	
114	11.65	2‐Furoic acid, hex‐4‐yn‐3‐yl ester	57	C_11_H_12_O_3_	1000299‐23‐5		✓
115	11.71	Linalool	98	C_10_H_18_O	78‐70‐6	✓	✓
116	11.80	2‐(1‐Hydroxy‐1‐methylethyl)pyrrolidine‐1‐carboxylic acid, methyl ester	64	C_9_H_17_NO_3_	1000187‐71‐5	✓	
117	11.82	5‐Hepten‐3‐one, 5‐methyl‐	66	C_8_H_14_O	1190‐34‐7		✓
118	11.84	1,2,3‐Propanetriol, 1‐acetate	96	C_5_H_10_O_4_	106‐61‐6		✓
119	11.95	2‐Furancarboxylic acid, 2‐tetrahydrofurylmethyl ester	63	C_10_H_12_O_4_	—	✓	
120	11.97	Ethyl 2,2‐diethoxypropionate	80	C_9_H_18_O_4_	7476‐20‐2	✓	
121	12.08	Maltol	70	C_6_H_6_O_3_	118‐71‐8	✓	✓
122	12.09	Phenylethyl alcohol	98	C8H_10_O	60‐12‐8	✓	
123	12.19	Benzenemethanol, .alpha.‐methyl‐	80	C_8_H_10_O	98‐85‐1		✓
124	12.30	4‐Methoxy‐*o*‐phenylenediamine	71	C_7_H_10_N_2_O	102‐51‐2	✓	
125	12.30	Benzene, 1,4‐dimethoxy‐	71	C_8_H_10_O_2_	150‐78‐7	✓	
126	12.69	Methyl isobutyl ketone	81	C_6_H_12_O	108‐10‐1		✓
127	12.73	Bicyclo[4.1.0]heptan‐3‐ol, 4,7,7‐trimethyl‐, [1*R*‐(1alpha,3alpha,4alpha,6alpha)]‐	79	C_10_H_18_O	4017‐89‐4	✓	
128	12.81	Acetic acid, cesium salt	56	C_2_H_3_CsO_2_	3396‐11‐0	✓	✓
129	13.05	Succinic acid, 3‐chlorophenyl 3‐phenylprop‐2‐en‐1‐yl ester	75	C_19_H_17_ClO_4_	1010391‐04‐7		✓
130	13.06	Cyclohexanol, 1‐methyl‐4‐(1‐methylethenyl)‐	91	C_10_H_18_O	138‐87‐4	✓	
131	13.22	Ethanethiol, 2‐(diethylboryloxy)‐	71	C_6_H_15_BOS	1000163‐05‐6	✓	✓
132	13.37	l‐Menthone	95	C_10_H_18_O	14073‐97‐3	✓	
133	13.49	Ethyl orthoformate	56	C_7_H_16_O_3_	122‐51‐0	✓	
134	13.49	2‐t‐Butyl‐6‐chloromethyl‐[1,3]dioxan‐4‐one	60	C_9_H_15_ClO_3_	139883‐58‐2	✓	
135	13.50	2,3‐Dicyano‐5,6‐diphenylpyrazine	58	C_18_H_10_N_4_	52197‐23‐6		✓
136	13.51	5‐Hydroxy‐7‐methoxy‐2‐methyl‐3‐phenyl‐4‐chromenone	78	C_17_H_14_O_4_	55927‐39‐4	✓	✓
137	13.60	Phthalic acid, 4‐cyanophenyl 2‐propyl ester	50	C_18_H_15_NO_4_	1000315‐57‐1		✓
138	13.69	2‐Butanol, 2,3‐dimethyl‐	69	C_6_H_14_O	594‐60‐5		✓
139	13.69	2,3‐Butanediol, 2,3‐dimethyl‐	58	C_0_H_14_O_2_	76‐09‐5	✓	
140	13.70	1,5‐Heptadiene, 2,3,6‐trimethyl‐	71	C_10_H_18_	33501‐88‐1		✓
141	13.75	Cyclohexanone, 5‐methyl‐2‐(1‐methylethyl)‐, *trans*‐	73	C_10_H_18_O	89‐80‐5	✓	
142	13.80	*N*‐1H‐Tetrazol‐5‐ylacetamide	54	C_3_H_5_N_5_O	6158‐77‐6	✓	
143	13.99	dl‐Menthol	83	C_10_H_20_O	89‐78‐1		✓
144	14.00	Methyl nitrite	92	CH_3_NO_2_	624‐91‐9	✓	✓
145	14.06	Trimethylaluminium	57	C_3_H_9_Al	75‐24‐1	✓	
146	14.07	Cyclohexanol, 5‐methyl‐2‐(1‐methylethyl)‐	99	C_10_H_20_O	1490‐04‐6	✓	
147	14.19	Diglycolic acid, isobutyl 3‐phenylpropyl ester	59	C_17_H_24_O_5_	1010382‐17‐1		✓
148	14.23	4‐Amino‐6‐hydroxypyrimidine	53	C_4_H_5_N_3_O	1193‐22‐2	✓	
149	14.25	Benzeneacetic acid, methyl ester	84	C_9_H_10_O_2_	101‐41‐7	✓	
150	14.31	Glycerine	91	C_3_H_8_O_3_	56‐81‐5	✓	✓
151	14.38	Azulene	58	C_10_H_8_	275‐51‐4	✓	
152	14.38	1,2‐Benzenedicarbonitrile	73	C_8_H_4_N_2_	91‐15‐6	✓	
153	14.54	Butanoic acid, 3‐hexenyl ester, (*Z*)‐	98	C_10_H_18_O_2_	16491‐36‐4	✓	✓
154	14.56	1,2,2‐Trimethylpropyl trifluoroacetate	61	C_8_H_13_F_3_O_2_	116465‐21‐5		✓
155	14.60	alpha‐Terpineol	83	C_10_H_18_O	98‐55‐5		✓
156	14.60	l‐alpha‐Terpineol	98	C_10_H_18_O	10482‐56‐1	✓	✓
157	14.84	*trans*‐5,6‐Dimethyl‐3,7,9‐trioxabicyclo[4.2.1]nonane	80	C_8_H_14_O_3_	31759‐27‐0		✓
158	14.84	2,3‐Pentanedione	78	C_5_H_8_O_2_	600‐14‐6		✓
159	14.86	Cyclohexanol, 1‐methyl‐4‐(1‐methylethylidene)‐	89	C_10_H_18_O	586‐81‐2	✓	
160	14.86	Cyclohexene, 3‐methyl‐6‐(1‐methylethylidene)‐	78	C_10_H_16_	586‐63‐0	✓	
161	14.88	4‐Pentenoic acid, 2,2‐diethyl‐3‐oxo‐5‐phenyl‐, ethyl ester	78	C_17_H_22_O_3_	337503‐48‐7	✓	
162	15.02	Succinic acid, tridec‐2‐yn‐1‐yl tetrahydrofurfuryl ester	56	C_22_H_36_O_5_	1000390‐72‐9		✓
163	15.18	Benzaldehyde, 2,4‐dihydroxy‐6‐methyl‐	83	C_8_H_8_O_3_	487‐69‐4	✓	
164	15.25	Dinocap	75	C_10_H_9_NO_4_	39300‐45‐3		✓
165	15.33	Cyclohexanol, 1‐(4‐fluorophenyl)‐4‐pentyl‐	56	C_17_H_25_FO	1000141‐78‐6	✓	
166	15.47	2‐[(4‐Fluorophenyl)methyl]‐5‐([(3‐methoxyphenyl)amino]methyl)‐2,3‐dihydro‐1*H*‐1,2,4‐triazol‐3‐one	57	C_17_H_17_FN_4_O_2_	1000386‐81‐5	✓	
167	15.50	1*H*‐Pyrazolo[3,4‐d]pyrimidin‐4‐amine	80	C_5_H_5_N_5_	2380‐63‐4		✓
168	15.50	Benzothiazole	78	C_7_H_5_NS	95‐16‐9		✓
169	15.65	2,6‐Octadien‐1‐ol, 3,7‐dimethyl‐, (*Z*)‐	94	C_10_H_18_O	106‐25‐2	✓	
170	15.66	Cyclohexane, isothiocyanato‐	89	C_7_H_11_NS	1122‐82‐3		✓
171	15.82	[1,3]Benzimidazo[2,1‐b]quinazolin‐1(2*H*)‐one, 3,4‐dihydro‐3,3‐dimethyl‐	52	C_16_H_15_N_3_O	1000318‐78‐3		✓
172	15.91	Pulegone	81	C_10_H_16_O	89‐82‐7	✓	
173	16.00	Carvone	78	C_10_H_14_O	99‐49‐0	✓	
174	16.02	Benzeneacetic acid, ethyl ester	83	C_10_H_12_O_2_	101‐97‐3	✓	
175	16.11	Caprolactam	90	C_6_H_11_NO	105‐60‐2	✓	
176	16.21	2,6‐Octadien‐1‐ol, 3,7‐dimethyl‐	87	C_10_H_18_O	624‐15‐7	✓	
177	16.21	2‐Propenoic acid, 2‐methyl‐, propyl ester	55	C_7_H_12_O_2_	2210‐28‐8		✓
178	16.23	Benzeneacetic acid, 2‐phenylethyl ester	87	C_16_H_16_O_2_	102‐20‐5		✓
179	16.27	2(3*H*)‐Furanone, 5‐acetyldihydro‐	72	C_6_H_8_O_3_	29393‐32‐6		✓
180	16.27	Acetic acid, 2‐phenylethyl ester	98	C_10_H_12_O_2_	103‐45‐7	✓	
181	16.29	2(3*H*)‐Furanone, 5‐butyldihydro‐	90	C_8_H_14_O_2_	104‐50‐7	✓	
182	16.34	Cyclobutaneacetonitrile, 1‐methyl‐2‐(1‐methylethenyl)‐	59	C_10_H_15_N	55760‐15‐1		✓
183	16.34	*N*‐(2,6‐Diethylphenyl)‐1,1,1‐trifluoromethane sulphonamide	51	C_11_H_14_F_3_NO_2_S	72846‐43‐6		✓
184	16.36	Hexanoic acid, 3‐phenyl‐2‐propenyl ester	54	C_15_H_20_O_2_	6994‐20‐3	✓	
185	16.52	Cinnamaldehyde, (*E*)‐	95	C_9_H_8_O	14371‐10‐9	✓	
186	16.57	1,2‐Dihydrolinalool	66	C_10_H_20_O	18479‐51‐1	✓	
187	16.65	*m*‐Ethylacetophenone	70	C_10_H_12_O	22699‐70‐3	✓	
188	16.68	Phenol, 4‐ethyl‐2‐methoxy‐	78	C_9_H_12_O_2_	2785‐89‐9	✓	
189	16.75	5‐Methyl‐2‐phenyl‐2‐hexenal	75	C_13_H_16_O	21834‐92‐4	✓	
190	16.75	Benzene, 1‐ethenyl‐4‐ethyl‐	52	C_10_H_12_	—	✓	
191	16.80	2‐Isopropenyl‐3,6‐dimethylpyrazine	72	C_9_H_12_N_2_	1000109‐60‐7	✓	
192	16.82	Oxazolidine, 2,2‐diethyl‐3‐methyl‐	64	C_8_H_17_NO	161500‐43‐2	✓	
193	16.90	1‐Methyl‐4‐isopropyl‐cyclohexyl 2‐hydroperfluorobutanoate	67	C_14_H_20_F_6_O_2_	1000145‐04‐8		✓
194	16.90	*Trans*‐3‐methyl‐4‐octanolide	97	C_9_H_16_O_2_	39638‐67‐0	✓	✓
195	16.92	4‐Hydroxy‐2‐methylacetophenone	78	C_9_H_10_O_2_	875‐59‐2		✓
196	16.94	(−)‐Neomenthyl acetate	69	C_12_H_22_O_2_	146502‐80‐9	✓	
197	16.95	4*H*‐1‐Benzopyran‐2‐carboxylic acid, 5‐amino‐6‐hydroxy‐4‐oxo‐, ethyl ester	53	C_12_H_11_NO_5_	32142‐43‐1		✓
198	17.13	2‐Propen‐1‐ol, 3‐phenyl‐	99	C_9_H_10_O	104‐54‐1	✓	✓
199	17.16	Naphthalene, 2‐methyl‐	73	C_11_H_10_	91‐57‐6		✓
200	17.19	2,6‐Difluorobenzoic acid, 3,5‐difluorophenyl ester	58	C_13_H_6_F_4_O_2_	1000292‐62‐1	✓	
201	17.38	2(3*H*)‐Furanone, 5‐butyldihydro‐4‐methyl‐	72	C_9_H_16_O_2_	39212‐23‐2		✓
202	17.40	2(3*H*)‐Furanone, 5‐butyldihydro‐4‐methyl‐, *cis*‐	98	C_9_H_16_O_2_	55013‐32‐6	✓	
203	17.42	Carbonic acid, monoamide, *N*‐decyl‐, benzyl ester	66	C_18_H_29_NO_2_	1000420‐29‐2	✓	
204	17.55	Piperonal	99	C_8_H_6_O_3_	120‐57‐0	✓	✓
205	17.71	Propanal, butylhydrazone	68	C_7_H_16_N_2_	20607‐75‐4	✓	
206	17.73	2,4,5,6,8‐Pentathianonane	53	C_4_H_10_S_5_	88496‐84‐8		✓
207	17.76	1,3‐Diacetin	98	C_7_H_12_O_5_	1000428‐18‐0		✓
208	17.77	1,3‐Cyclohexadiene, 1‐methyl‐4‐(1‐methylethyl)‐	76	C_10_H_16_	99‐86‐5	✓	
209	17.77	Glycerol 1,2‐diacetate	98	C_7_H_12_O_5_	102‐62‐5	✓	✓
210	17.89	(2*E*)‐*N*‐[2‐(3,4‐Dimethoxyphenyl)ethyl]‐*N*‐methyl‐3‐phenyl‐2‐propenamide	65	C_20_H_23_NO_3_	193901‐52‐9	✓	
211	17.92	(*E*)‐3,7‐Dimethylocta‐2,6‐dienyl ethyl carbonate	90	C_13_H_22_O_3_	1000373‐76‐7		✓
212	17.95	2,6‐Octadien‐1‐ol, 3,7‐dimethyl‐, acetate, (*Z*)‐	98	C_12_H_20_O_2_	141‐12‐8	✓	
213	17.96	6,7‐Dimethoxyquinoxaline	68	C_10_H_10_N_2_O_2_	6295‐29‐0	✓	
214	18.11	3‐Methoxyformanilide	56	C_8_H_9_NO_2_	27153‐17‐9		✓
215	18.11	1,2‐Benzenediol, *o*‐(4‐butylbenzoyl)‐*o*′‐(2‐methylbenzoyl)‐	73	C_25_H_24_O_4_	1000325‐96‐0		✓
216	18.14	Acenaphthene	81	C_12_H_10_	83‐32‐9		✓
217	18.14	*p*‐*tert*‐Octylresorcinol	64	C_14_H_22_O_2_	28122‐52‐3	✓	
218	18.15	Bicyclosesquiphellandrene	79	C_15_H_24_	54324‐03‐7	✓	
219	18.15	(1*S*,4*S*,4*aS*)‐1‐Isopropyl‐4,7‐dimethyl‐1,2,3,4,4a,5‐hexahydronaphthalene	69	C_15_H_24_	267665‐20‐3	✓	
220	18.19	*o*‐Ethylhydroxylamine	60	C_2_H_7_NO	624‐86‐2		✓
221	18.20	Geranyl acetate	98	C_12_H_20_O_2_	105‐87‐3	✓	
222	18.26	2‐Buten‐1‐one, 1‐(2,6,6‐trimethyl‐1,3‐cyclohexadien‐1‐yl)‐, (*E*)‐	95	C_13_H_18_O	23726‐93‐4	✓	✓
223	18.29	2‐Buten‐1‐one, 1‐(2,6,6‐trimethyl‐1‐cyclohexen‐1‐yl)‐	75	C_13_H_20_O	35044‐68‐9	✓	
224	18.36	2‐Buten‐1‐one, 1‐(2,6,6‐trimethyl‐2‐cyclohexen‐1‐yl)‐, (*E*)‐	92	C_13_H_20_O	24720‐09‐0	✓	✓
225	18.39	Pyrrolidine‐2,5‐dione, 1‐(2‐nitro‐3‐pyridyl)‐	53	C_9_H_7_N_3_O_4_	123494‐96‐2	✓	
226	18.45	Benzaldehyde, 3‐hydroxy‐4‐methoxy‐	82	C_8_H_8_O_3_	621‐59‐0	✓	
227	18.45	Vanillin	84	C_8_H_8_O_3_	121‐33‐5	✓	
228	18.47	Cyclohexanol, 3‐(3,3‐dimethylbutyl)‐	57	C_12_H_24_O	40564‐98‐5		✓
229	18.47	3‐Hexen‐1‐ol, propanoate, (*Z*)‐	72	C_9_H_16_O_2_	33467‐74‐2		✓
230	18.51	(*E*)‐beta‐Farnesene	76	C_15_H_24_	18794‐84‐8		✓
231	18.51	2‐Furancarboxylic acid, hexyl ester	61	C_11_H_16_O_3_	39251‐86‐0	✓	
232	18.56	Benzene, (1‐methyl‐1‐propylpentyl)‐	65	C_15_H_24_	54932‐91‐1	✓	
233	18.56	Benzene, 1,3‐dimethyl‐	60	C_8_H_10_	108‐38‐3	✓	
234	18.57	Butanoic acid, 3,7‐dimethyl‐2,6‐octadienyl ester, (*E*)‐	67	C_14_H_24_O_2_	106‐29‐6	✓	
235	18.59	Damascone, beta‐	98	C_13_H_20_O	23726‐91‐2		✓
236	18.67	Bicyclo[5.2.0]nonane, 2‐methylene‐4,8,8‐trimethyl‐4‐vinyl‐	95	C_15_H_24_	242794‐76‐9		✓
237	18.65	Benzeneacetic acid, 4‐methoxy‐, methyl ester	64	C_10_H_12_O_3_	23786‐14‐3	✓	
238	18.65	Benzenemethanol, 4‐methoxy‐, acetate	70	C_10_H_12_O_3_	104‐21‐2	✓	
239	18.67	4,8‐Dimethylnona‐1,3,7‐triene	70	C_11_H_18_	51911‐82‐1	✓	
240	18.72	Caryophyllene	89	C_15_H_24_	87‐44‐5	✓	
241	18.72	Naphthalene, 1,2,3,5,6,7,8,8*a*‐octahydro‐1,8*a*‐dimethyl‐7‐(1‐methylethenyl)‐, [1*R*‐(1alpha,7beta,8a alpha)]‐	71	C_15_H_24_	—	✓	
242	18.81	1,2,4‐Triazolo[4,3‐a]pyridine‐3(2*H*)‐thione	59	C_6_H_5_N_3_S	6952‐68‐7	✓	
243	18.82	2‐Propanone, 1,1,1,3‐tetrachloro‐	56	C_3_H_2_Cl_4_O	16995‐35‐0	✓	
244	18.82	Cycloocta‐1,3,6‐triene, 2,3,5,5,8,8‐hexamethyl‐	60	C_14_H_22_	1000161‐97‐9	✓	
245	18.82	2‐Acetyl‐4‐methylbenzo(b)thiophene	72	C_11_H_10_OS	1467‐88‐5	✓	
246	18.87	Geranyl isobutyrate	60	C_14_H_24_O_2_	2345‐26‐8	✓	
247	18.94	Acetic acid, cinnamyl ester	98	C_11_H_12_O_2_	103‐54‐8	✓	✓
248	18.96	3,6‐Dimethoxy‐1*a*,2,2*a*,3,6,6*a*,7,7*a*‐octahydro‐1‐oxacyclopropa[*b*]naphthalene	54	C_12_H_18_O_3_	1010191‐51‐4	✓	
249	19.07	Ethyl vanillin	94	C_9_H_10_O_3_	121‐32‐4	✓	
250	19.10	1,4,7,‐Cycloundecatriene, 1,5,9,9‐tetramethyl‐, *Z,Z,Z*‐	82	C_15_H_24_	1000062‐61‐9	✓	✓
251	19.15	Decane, 1‐chloro‐	64	C_10_H_21_Cl	1002‐69‐3		✓
252	19.17	2‐Propenoic acid, 3‐phenyl‐, ethyl ester	91	C_11_H_12_O_2_	103‐36‐6	✓	
253	19.18	Cyclohexanol, 1‐ethenyl‐	61	C_8_H_14_O	1940‐19‐8		✓
254	19.18	Cyclobutane, 1,2:3,4‐di‐*O*‐ethylboranediyl‐	55	C_8_H_14_B_2_O_4_	1000159‐65‐2		✓
255	19.21	2(3*H*)‐Furanone, 5‐hexyldihydro‐	97	C_10_H_18_O_2_	706‐14‐9	✓	✓
256	19.29	Benzene, 1,1′‐[1,5‐pentanediylbis(oxymethylene)]bis‐	64	C_19_H_24_O_2_	53150‐24‐6	✓	
257	19.32	Benzaldehyde, 3,4‐dimethoxy‐	75	C_9_H_10_O_3_	120‐14‐9	✓	
258	19.34	1,3‐Cyclohexanedione, 2‐[2‐[4‐(4‐fluorobenzoyl)‐1‐piperazinyl]ethylaminomethylene]‐	66	C_22_H_28_FN_3_O_3_	339310‐26‐8	✓	
259	19.38	*trans*‐beta‐Ionone	95	C_13_H_20_O	79‐77‐6		✓
260	19.41	3‐Buten‐2‐one, 4‐(2,6,6‐trimethyl‐1‐cyclohexen‐1‐yl)‐	98	C_13_H_20_O	14901‐07‐6	✓	
261	19.42	2‐(2‐Naphthyl)‐2‐propanol	59	C_13_H_14_O	20351‐54‐6	✓	
262	19.47	Cycloheptasiloxane, tetradecamethyl‐	97	C_14_H_42_O_7_Si_7_	107‐50‐6	✓	
263	19.49	2*H*‐Pyran‐2‐one, tetrahydro‐6‐propyl‐	85	C_8_H_14_O_2_	698‐76‐0	✓	
264	19.59	3,4‐Dimethyl‐2‐(3‐methyl‐butyryl)‐benzoic acid, methyl ester	60	C_15_H_20_O_3_	71940‐29‐9		✓
265	19.62	Pentanoic acid, 5‐hydroxy‐, 2,4‐di‐*t*‐butylphenyl esters	87	C_19_H_30_O_3_	166273‐38‐7	✓	
266	19.62	Pyrolo[3,2‐d]pyrimidin‐2,4(1*H*,3*H*)‐dione	69	C_6_H_5_N_3_O_2_	65996‐50‐1	✓	
267	19.64	Decahydronaphtho[2,3‐*b*]furan‐2‐one, 3‐[(4‐methoxybenzylamino)methyl]‐8*a*‐methyl‐5‐methylene‐	67	C_23_H_31_NO_3_	1010302‐53‐3		✓
268	19.67	1,3‐Pentadiyne, 1,5,5,5‐tetrafluoro‐	58	C_5_F_4_	64788‐24‐5	✓	
269	19.68	2‐(1‐Benzyloxy‐2‐bromoethyl)oxirane	69	C_11_H_13_BrO_2_	101514‐16‐3	✓	
270	19.68	Oxirane, 2‐methyl‐3‐[(phenylmethoxy)methyl]‐	69	C_11_H_14_O_2_	116296‐88‐9	✓	
271	19.75	Carbonic acid, methyl ester, [(*E*)‐3,7‐dimethyl‐2,6‐octadien‐1‐yl] ester	80	C_12_H_20_O_3_	85217‐72‐7	✓	
272	19.79	1‐Penten‐3‐one, 1‐(4‐methoxyphenyl)‐4‐methyl‐	67	C_13_H_16_O_2_	103‐13‐9	✓	
273	19.79	Naphthalene, 1,2,3,5,6,8a‐hexahydro‐4,7‐dimethyl‐1‐(1‐methylethyl)‐, (1*S*‐*cis*)‐	51	C_15_H_24_	483‐76‐1	✓	
274	19.82	4‐(1,2‐Dimethyl‐cyclopent‐2‐enyl)‐butan‐2‐one	61	C_11_H_18_O	75698‐06‐5	✓	
275	19.88	4‐Hexen‐1‐ol, 5‐methyl‐2‐(1‐methylethenyl)‐, (*R*)‐	80	C_10_H_18_O	498‐16‐8	✓	✓
276	19.93	5‐Fluoro‐2‐trifluoromethylbenzoic acid, 4‐nitrophenyl ester	64	C_14_H_7_F_4_NO_4_	1000357‐63‐6		✓
277	19.95	Hexanoic acid, phenylmethyl ester	71	C_13_H_18_O_2_	6938‐45‐0	✓	
278	19.97	4‐sec‐Butylphenol, *o*‐ethoxycarbonyl‐	64	C_13_H_18_O_3_	1000487‐30‐7		✓
279	20.11	Bicyclo[2.2.2]oct‐2‐ene, 1,2,3,6‐tetramethyl‐	82	C_12_H_20_	62376‐14‐1	✓	
280	20.22	2(3*H*)‐Furanone, 5‐heptyldihydro‐	94	C_11_H_20_O_2_	104‐67‐6	✓	
281	20.25	1‐(1,2‐Dimethoxypropyl)‐4‐methoxybenzene	58	C_12_H_18_O_3_	138169‐72‐9	✓	
282	20.27	Carbazole, tetrahydro‐9‐acetyl‐	64	C_14_H_15_NO	27236‐49‐3	✓	
283	20.28	*N*‐(3‐Pyridinyl)‐2‐thiophenecarboxamide	52	C_10_H_8_N_2_OS	62289‐81‐0	✓	
284	20.36	Cyclohexanecarboxylic acid, 4‐propyl‐, 4‐ethoxyphenyl ester, *trans*‐	68	C_18_H_26_O_3_	67589‐39‐3	✓	
285	20.37	Cyclopenten‐4‐one, 1,2,3,3‐tetramethyl‐	61	C_9_H_14_O	1000163‐38‐6	✓	
286	20.38	10,10‐Dimethyl‐2,6‐dimethylenebicyclo[7.2.0]undecane	55	C_15_H_24_	357414‐37‐0		✓
287	20.40	Caryophyllene oxide	95	C_15_H_24_O	1139‐30‐6	✓	
288	20.43	(4‐Methylbenzo(b)thien‐2‐yl)acetamide	51	C_11_H_11_NOS	1000244‐51‐7	✓	
289	20.43	5,9‐Undecadien‐1‐yne, 6,10‐dimethyl‐	56	C_13_H_20_	100451‐98‐7		✓
290	20.54	Humulene epoxide I	75	C_15_H_24_O	19888‐33‐6	✓	
291	20.62	Butylphosphonic acid, di(4‐octyl) ester	54	C_20_H_43_O_3_P	1000322‐96‐0		✓
292	20.65	Methyl 5‐(1,2,4‐triazol‐1‐ylmethyl)furan‐2‐carboxylate	65	C_9_H_9_N_3_O_3_	1000388‐08‐9	✓	
293	20.71	2‐Methyl‐2‐adamantanol	63	C_11_H_18_O	702‐98‐7	✓	
294	20.77	Benzophenone	92	C_13_H_10_O	119‐61‐9	✓	
295	20.82	Phosphoryl fluoride	51	F_3_OP	13478‐20‐1	✓	
296	20.82	2‐Ethoxyamphetamine	67	C_11_H_17_NO	135014‐84‐5		✓
297	20.86	10,10‐Dimethyl‐2,6‐dimethylenebicyclo[7.2.0]undecan‐5beta‐ol	93	C_15_H_24_O	19431‐80‐2	✓	
298	20.89	2‐Furancarboxylic acid, octyl ester	80	C_13_H_20_O_3_	39251‐88‐2	✓	
299	21.02	14‐Hydroxycaryophyllene	93	C_15_H_24_O	50277‐33‐3	✓	
300	21.02	Tetradecane, 1‐chloro‐	69	C_14_H_29_Cl	2425‐54‐9		✓
301	21.07	1,4‐Dimethyltricyclo[5.3.0.0(4.10)]decan‐8‐one	67	C_12_H_18_O	138041‐97‐1	✓	
302	21.07	4,7‐Methanobenzofuran, 2,2′‐oxybis[octahydro‐7,8,8‐trimethyl‐, [2alpha (2′R*,3′aS*,4′R*,7′R*,7′aS*),3*a* alpha,4alpha,7alpha,7*a* alpha]‐	64	C_24_H_38_O_3_	81955‐10‐4	✓	
303	21.11	2‐Methyl‐5‐[2‐(5‐methylthiophen‐2‐yl)ethyl]thiophene	50	C_12_H_14_S_2_	1000487‐09‐5	✓	
304	21.38	2*H*‐Pyran‐2‐one, tetrahydro‐6‐pentyl‐	90	C_10_H_18_O_2_	705‐86‐2	✓	
305	21.41	1,2‐Ethanediol, monoformate	79	C_3_H_6_O_3_	628‐35‐3		✓
306	21.46	3‐Methoxy‐5‐methylphenol	57	C_8_H_10_O_2_	3209‐13‐0	✓	
307	21.53	Hydrazine, (2‐methoxyethyl)‐	57	C_3_H_10_N_2_O	3044‐15‐3		✓
308	21.53	2‐[2‐[2‐[2‐[2‐[2‐[2‐(2‐Hydroxyethoxy)ethoxy]ethoxy]ethoxy]ethoxy]ethoxy]ethoxy]ethanol	54	C_16_H_34_O_9_	5117‐19‐1		✓
309	21.59	1,2‐Benzenediol, *o*‐(2‐bromopropionyl)‐*o*′‐(4‐ethylbenzoyl)‐	68	C_18_H_17_BrO_4_	1000325‐97‐0		✓
310	21.60	4‐Ethylbenzoic acid, 2,3‐dichlorophenyl ester	52	C_15_H_12_Cl_2_O_2_	1000331‐31‐5		✓
311	21.65	Octanoic acid, phenylmethyl ester	94	C_15_H_22_O_2_	10276‐85‐4	✓	
312	21.67	Tetradecanoic acid	84	C_14_H_28_O_2_	544‐63‐8		✓
313	21.89	Ambrox	97	C_16_H_28_O	100679‐85‐4	✓	✓
314	22.03	Adamantane‐1‐carboxamide, *N*‐[2‐(3,4‐dimethylphenoxy)ethyl]‐	61	C_21_H_29_NO_2_	329731‐40‐0	✓	
315	22.09	2‐Ethylhexyl salicylate	55	C_15_H_22_O_3_	118‐60‐5		✓
316	22.14	2‐sec‐Butylphenol, *o*‐ethoxycarbonyl‐	73	C_13_H_18_O_3_	1000487‐26‐4	✓	
317	22.23	1,2‐Cyclohexanediol, 1‐methyl‐4‐(2‐methyl‐1,3‐dioxolan‐2‐yl)‐	58	C_11_H_20_O_4_	56859‐97‐3	✓	
318	22.25	Fumaric acid, 2,6‐dimethoxyphenyl 3‐methylbut‐2‐en‐1‐yl ester	61	C_17_H_20_O_6_	1000405‐75‐1	✓	
319	22.29	5‐(3,3‐Dimethylbicyclo[2.2.1]heptan‐2‐yl)pent‐3‐en‐2‐one (isomer 2)	67	C_14_H_22_O	1000497‐97‐8	✓	
320	22.33	3,4‐Dihydrocoumarin, 4,4‐dimethyl‐6‐hydroxy‐	57	C_11_H_12_O_3_	29423‐72‐1		✓
321	22.33	Phenol, 5‐methyl‐2‐(1‐methylethyl)‐, acetate	68	C_12_H_16_O_2_	528‐79‐0		✓
322	22.36	2‐Amino‐2,4,6‐cycloheptatriene‐1‐thione	57	C_7_H_7_NS	3336‐99‐0	✓	
323	22.37	3beta,9beta‐Dihydroxy‐3,5alpha,8‐trimethyltricyclo[6.3.1.0(1,5)]dodecane	52	C_15_H_26_O_2_	1000140‐34‐8	✓	
324	22.39	Pentadecanoic acid	75	C_15_H_30_O_2_	1002‐84‐2		✓
325	22.40	*N*‐[2‐(4‐Chloro‐phenoxy)‐acetyl]‐*N*′‐(4,7‐dimethyl‐quinazolin‐2‐yl)‐guanidine	54	C_19_H_18_ClN_5_O_2_	1000275‐15‐1		✓
326	22.42	3,3′‐Dimenthol	55	C_20_H_38_O_2_	148552‐94‐7	✓	
327	22.48	Benzene, 1‐(4‐morpholylcarbonyl)‐4‐[2‐(4‐tolylthio)ethoxy]‐	58	C_20_H_23_NO_3_S	1000258‐53‐1	✓	
328	22.51	Bis(2‐hydroxyethyl) phthalate, acetate	54	C_14_H_1_6O_7_	1010503‐08‐1		✓
329	22.54	Cyclohexane, 1‐ethyl‐2‐propyl‐	73	C_11_H_22_	62238‐33‐9		✓
330	22.55	*S*‐Methyl pentanethioate	59	C_6_H_12_OS	42075‐43‐4		✓
331	22.55	Phthalic acid, 6‐ethyl‐3‐octyl butyl ester	68	C_22_H_34_O_4_	1000315‐17‐4	✓	
332	22.68	Thiophene‐2‐carboxamide, *N*‐(3‐chlorophenyl)‐	50	C_11_H_8_ClNOS	1000307‐07‐0	✓	
333	22.76	Caffeine	80	C_8_H_10_N_4_O_2_	58‐08‐2		✓
334	22.85	Octane, 1,1‐diethoxy‐	56	C_12_H_26_O_2_	54889‐48‐4		✓
335	22.85	Phthalic acid, ethyl tridec‐2‐yn‐1‐yl ester	70	C_23_H_32_O_4_	1000315‐43‐5	✓	
336	22.91	1,3‐Dioxolane, 2‐heptyl‐4‐methyl‐	78	C_11_H_22_O_2_	74094‐61‐4	✓	
337	22.96	Hexadecenoic acid, *Z*‐11‐	81	C_16_H_30_O_2_	2416‐20‐8		✓
338	22.96	Cyclohexane, 1‐(cyclohexylmethyl)‐3‐methyl‐, *cis*‐	77	C_14_H_26_	54823‐96‐0		✓
339	22.96	1,2,3,4,5,6,7,8‐Octahydro‐2‐naphthol, 4‐methylene‐2,5,5‐trimethyl‐	54	C_14_H_22_O	125257‐67‐2	✓	
340	22.97	4‐[1,3]Dioxan‐2‐yl‐3,4‐dimethylcyclohex‐2‐enone	79	C_12_H_18_O_3_	1000191‐48‐2	✓	
341	23.10	*n*‐Hexadecanoic acid	97	C_16_H_32_O_2_	57‐10‐3		✓
342	23.46	Sulphurous acid, hexyl 2‐pentyl ester	63	C_11_H_24_O_3_S	1000309‐15‐6		✓
343	23.46	1‐Nonene, 4,6,8‐trimethyl‐	69	C_12_H_24_	54410‐98‐9		✓
344	23.61	Cyclopentane, 1,1,3‐trimethyl‐3‐(2‐methyl‐2‐propenyl)‐	59	C_12_H_22_	74421‐09‐3		✓
345	23.74	*Myo*‐Inositol, 4‐C‐methyl‐	59	C_7_H_14_O_6_	472‐95‐7		✓
346	23.74	Dodecanoic acid	57	C_12_H_24_O_2_	143‐07‐7		✓
347	23.89	Cyclooctane, 1‐methyl‐3‐propyl‐	78	C_12_H_24_	255885‐37‐1		✓
348	24.00	5‐Methyl‐2,4‐diisopropylphenol	60	C_13_H_20_O	40625‐96‐5		✓
349	24.08	2‐Pentoxy‐tetrahydropyran	62	C_10_H_20_O_2_	32767‐70‐7		✓
350	24.24	18‐Nonadecen‐1‐ol	80	C_19_H_38_O	1000142‐89‐2		✓
351	24.24	Oleic acid	81	C_18_H_34_O_2_	112‐80‐1		✓
352	24.37	Octadecanoic acid	89	C_18_H_36_O_2_	57‐11‐4		✓
353	24.58	Bicyclo[1.1.1]pentane, 1,3‐dipropanoyl‐	53	C_11_H_16_O_2_	1000287‐90‐9	✓	
354	24.66	Phenol, 4,4′‐(1‐methylethylidene)bis‐	74	C_15_H_16_O_2_	80‐05‐7		✓
355	24.80	1‐Buten‐1‐amine, *N,N*‐dipropyl‐	62	C_10_H_21_N	88557‐03‐3		✓
356	25.16	Decane, 2,3,8‐trimethyl‐	75	C_13_H_28_	62238‐14‐6		✓
357	25.71	(1*S*,4*aR*,5*S*,8*aR*)‐1,4*a*‐Dimethyl‐6‐methylene‐5‐((*E*)‐3‐methylpenta‐2,4‐dien‐1‐yl)decahydronaphthalene‐1‐carboxylic acid	74	C_20_H_30_O_2_	2761‐77‐5		✓
358	26.22	1‐Phenanthrenecarboxylic acid, 1,2,3,4,4*a*,9,10,10*a*‐octahydro‐1,4*a*‐dimethyl‐7‐(1‐methylethyl)‐, [1*S*‐(1alpha,4*a* alpha,10*a* beta)]‐	68	C_20_H_28_O_2_	5155‐70‐4		✓
359	26.83	Phthalic acid, di(hept‐4‐yl) ester	73	C_22_H_34_O_4_	1000356‐80‐0		✓
360	27.72	1‐((1‐Butoxypropan‐2‐yl)oxy)propan‐2‐yl 2,3,4,5,6‐pentafluorobenzoate	53	C_17_H_21_F_5_O_4_	1000378‐29‐1	✓	
361	27.82	Naphthalene, 1‐(2‐naphthalenyloxy)‐	63	C_20_H_14_O	611‐49‐4		✓
362	28.38	26‐Nor‐5‐cholesten‐3beta‐ol‐25‐one	75	C_26_H_42_O_2_	7494‐34‐0		✓
363	28.39	*N*‐{4‐[4‐(4‐Azido‐1,2,5‐oxadiazol‐3‐yl)‐1,2,5‐oxadiazol‐3‐yl]‐1,2,5‐oxadiazol‐3‐yl}‐*N*‐methylacetamide	58	C_9_H_6_N_10_O_4_	1000459‐49‐6		✓
364	28.85	3‐(*t*‐Octylamino)propionitrile	66	C_11_H_22_N_2_	86375‐28‐2		✓
365	28.85	Oxalic acid, cyclobutyl heptadecyl ester	67	C_23_H_42_O_4_	1000309‐70‐7		✓
366	28.86	(2*S*)‐2‐(2‐Furoylamino)‐4‐methylpentanoic acid, *N*‐methyl‐, methyl ester	56	C_13_H_19_NO_4_	1000505‐38‐8		✓
367	29.87	2,2‐Dimethylpropanoic acid, oct‐3‐en‐2‐yl ester	74	C_13_H_24_O_2_	1000299‐33‐3		✓
368	30.73	Methyl 3‐bromo‐1‐adamantaneacetate	56	C_13_H_19_BrO_2_	14575‐01‐0		✓
369	31.37	2‐Thiophenethiol	51	C_4_H_4_S_2_	7774‐74‐5		✓
370	33.23	Propane, 1,1‐dibromo‐2‐chloro‐	60	C_3_H_5_Br_2_Cl	55162‐35‐1		✓
371	33.40	l‐Proline, 1‐(trifluoroacetyl)‐, 1‐methylpropyl ester, (*R*)‐	53	C_11_H_16_F_3_NO_3_	55056‐63‐8		✓
372	33.40	2‐Propenoic acid, 2‐methyl‐, octyl ester	52	C_12_H_22_O_2_	9/01/2157		✓
373	33.43	2,2,4,5‐Tetramethyl‐5‐hexen‐3‐one	60	C_10_H_18_O	1000424‐66‐4		✓
374	33.47	2,4‐Diamino‐8‐hydroxy‐5‐(pentan‐3‐yl)‐5*H*‐chromeno[2,3‐*b*]pyridine‐3‐carbonitrile	60	C_18_H_20_N_4_O_2_	1000444‐37‐4		✓
375	34.72	Benzamide, 2‐methoxy‐*N*‐(2‐ethylphenyl)‐	59	C_16_H_17_NO_2_	1000339‐10‐9		✓

In the e‐liquid sample, several terpenes and flavour‐related substances were identified, including d‐limonene, l‐menthone, l‐α‐terpineol, carvone and pulegone, all commonly associated with essential oils and widely used as flavouring agents in consumer products. Distinctly, pulegone is classified as a potentially toxic compound and was included in a report by the US Food and Drug Administration (FDA) recommending the removal of seven synthetic flavouring substances from the food additives list [55]. Other compounds detected in this study, such as myrcene and benzophenone, were also part of that FDA report due to their toxicological concerns. Pulegone has demonstrated hepatotoxic and carcinogenic properties in animal studies, raising significant safety concerns when inhaled chronically [56].

The detection of toluene, a known VOC with toxic and irritant properties, raises concerns about the potential health risks associated with repeated exposure to these aerosols. Although other BTEX compounds such as ethylbenzene and xylene isomers were not detected in the present sample, the presence of even a single representative of this group highlights the necessity of further toxicological evaluation of e‐liquid formulations. In this sense, previous studies analysing commercial e‐liquids and aerosols have reported the occurrence of the full BTEX group [17, 32]. Additionally, compounds such as maltol and vanillin were identified, which are widely used for their sweet, caramel or vanilla‐like aroma profiles. The presence of glycerine (glycerol), glycidol, a common base solvent in e‐liquid formulations, was also confirmed, further indicating the typical composition of commercial vaping products. Other toxicologically relevant compounds were also found, including phenols, methylated alkylbenzenes, and PAHs such as azulene and naphthalene derivatives, all of which are associated with irritant, neurotoxic or potentially mutagenic effects [57].

**FIGURE 4:**
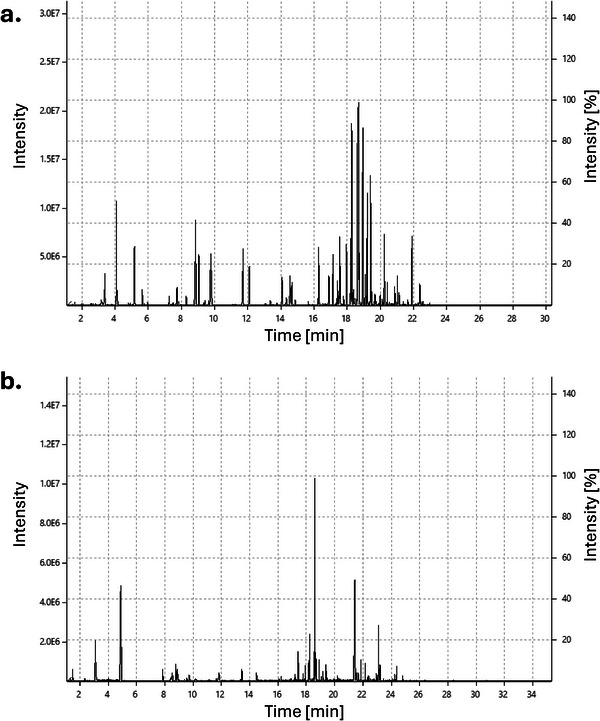
Comparative chromatograms obtained by SPME–GC–MS: (a) direct immersion (DI‐SPME) analysis of the e‐liquid; (b) in situ analysis of the aerosol, showing an increased presence of semi‐volatile compounds and late‐eluting degradation products, which required an extended analysis time of 4 min to be detected.

The chemical profile obtained from the in situ SPME–GC–MS analysis of the aerosol revealed a markedly different composition compared to the e‐liquid, with only a small subset of compounds shared between both matrices. The heating process involved in aerosol generation not only facilitates the transfer of volatile compounds from the e‐liquid but also induces thermal degradation and chemical transformations that may lead to the formation of new substances [58, 59]. In some cases, this process results in the emergence of harmful by‐products, such as PAHs and reactive aldehydes, which are associated with carcinogenicity, respiratory irritation and oxidative stress [60, 61]. Analysis of the chromatographic profiles further supports these observations: As shown in Figure 4, the e‐liquid (Figure 4a) displays a pattern dominated by more volatile constituents, whereas the aerosol (Figure 4b) exhibits a greater number of peaks towards the end of the run, indicative of compounds with higher boiling points and semi‐volatile characteristics. Consistent with the data summarized in Table 3, the aerosol profile required an additional 4 min of analysis time to ensure that late‐eluting compounds were captured and not overlooked.

From a toxicological perspective, in addition to the detection of naphthalene derivatives, acenaphthene was also identified, a compound known to cause liver toxicity in some organisms [62]. Additionally, the identification of dinocap, a fungicide with established toxicological significance, further emphasizes the potential health risks posed by exposure to these aerosols [63]. Phthalic acids were also detected in both the e‐liquid (phthalic acid, 6‐ethyl‐3‐octyl butyl ester, and phthalic acid, ethyl tridec‐2‐yn‐1‐yl ester) and aerosol matrices (phthalic acid, 4‐cyanophenyl 2‐propyl ester, and phthalic acid, di(hept‐4‐yl) ester), indicating persistence through the vaporization process. These compounds, widely used as intermediates in the production of plasticizers, have been associated with endocrine‐disrupting effects, reproductive toxicity and developmental abnormalities following chronic exposure [64, 65, 66]. Compounds such as pulegone and toluene, previously detected in the e‐liquid, were not found in the aerosol, suggesting they may have degraded or been lost during vaporization.

Under certain thermal conditions, however, other compounds may also be formed, some of which have been described in scientific literature as having anti‐inflammatory, antibacterial or antifungal properties. For instance, benzothiazole, a heterocyclic aromatic compound detected in the aerosol in this study, has been reported to possess such biological activities. Studies have further explored its potential as a precursor in drug development, particularly in the design of central nervous system (CNS) modulators and enzyme inhibitors [67, 68]. Nevertheless, the presence of such compounds in e‐cigarette aerosols does not necessarily imply therapeutic benefit upon inhalation, as factors such as bioavailability, exposure dose and interactions with other aerosol constituents remain unclear. Therefore, although the formation of benzothiazole illustrates the complex chemistry involved in e‐cigarette emissions, the overall health impact remains uncertain and warrants further investigation.

## 4 Perspectives

This study highlights the value of integrating epidemiological data with chemical analysis to provide a more comprehensive understanding of ESD use. By combining population‐level behavioural patterns with detailed chemical profiling, such investigations offer a holistic view of both the prevalence and potential toxicological implications of this habit, particularly among young adults. This interdisciplinary approach bridges the gap between perceived risk and actual exposure, reinforcing the importance of evidence‐based public health interventions.

To deepen our knowledge of the chemical risks associated with ESDs, future studies should incorporate more advanced analytical instrumentation capable of identifying and quantifying additional harmful compounds, including heavy metals, TSNAs and vitamin E acetate, substances increasingly reported in the scientific literature for their potential adverse effects on respiratory health.

Furthermore, the dissemination of findings from these studies should not be limited to academic platforms. Effective science communication strategies must include outreach through social media channels and other digital platforms frequently used by young people. This broader communication effort will enhance public awareness and support behavioural change, reinforcing prevention strategies at the societal level.

## 5 Conclusions

This study identified a high prevalence of ESD use and early signs of nicotine dependence among university students in Salvador, Brazil. Chemical analysis of e‐liquids and generated aerosols revealed a complex mixture of solvents, flavouring agents and toxicologically relevant compounds such as pulegone, myrcene, PAH derivatives and fungicide dinocap. These findings highlight the chemical complexity and potential health risks of ESD emissions, underscoring the need for continued chemical monitoring and targeted public health interventions.

## Author Contributions

**Eduard F. Valenzuela** and **Ivana Ferreira Simões**: conceptualization and execution of the chemical profiling analysis of the e‐liquid and aerosol, data interpretation, writing – original draft preparation. **Raffael Silva Santos Almeida**, **Magno Oliveira Ramos** and **Aline Gonçalves Miranda**: coordination and execution of interviews, statistical data collection, curation, and analysis. **Fernanda Warken Rosa**, **Roberto Rodrigues Bandeira Tosta Maciel** and **Aníbal de Freitas Santos Júnior**: writing – review and editing, supervision of the study and critical revision of the manuscript for important intellectual content.

## Conflicts of Interest

The authors declare no conflicts of interest.

## Data Availability

The data supporting the findings of this study are available from the corresponding author upon reasonable request.
